# Initial blood culture collection practices and the associated factors upon continued empiric piperacillin-tazobactam usage

**DOI:** 10.1017/ash.2023.257

**Published:** 2023-09-29

**Authors:** Satoshi Kitaura, Koh Okamoto, Ryo Yamaguchi, Takehito Yamamoto, Toshiyuki Kishida, Daisuke Inoue, Hirotaka Miyashita, Masayuki Ueda, Hideki Hashimoto, Sohei Harada, Shu Okugawa, Takeya Tsutsumi, Kyoji Moriya

## Abstract

**Background:** Approaches to the prescription behavior of broad-spectrum antibiotics, including preauthorization and prospective audit and feedback (PAF), are a focus of antimicrobial stewardship (ASP). However, preprescription behavior, such as blood-culture collection before empiric prescription, is understudied and merits more attention given its influence on the usage of broad-spectrum antibiotics. At the University of Tokyo Hospital, carbapenems are subject to PAF, which has resulted in a compensatory increase in piperacillin-tazobactam use. To evaluate the inherent preprescription behavior associated with a broad-spectrum antibiotic, we investigated the initial blood-culture collection practices upon hospitalization in patients who were continued on empiric piperacillin-tazobactam. **Methods:** A retrospective observational study was conducted at the University of Tokyo Hospital, a tertiary-care hospital in Tokyo, Japan. Patients who were administered piperacillin-tazobactam on the day of hospitalization between April 2016 and December 2017 were included. Patients aged <=18 years and/or patients who discontinued piperacillin-tazobactam within two days were excluded. Only 1 admission per patient was kept for analysis. The medical records of 250 randomly selected patients were reviewed to obtain data on demographics, blood-culture collection, severity, specialties, and risk factors for multidrug-resistant organisms. A multivariable logistic regression analysis was used to identify factors associated with blood-culture collection. **Results:** In total, 960 discrete patients fulfilled the study criteria. Of the randomly selected 250 patients, blood cultures were collected from 162 patients (64.8%), and microbial growth was observed in 30 cases (18.5%). Enterobacterales and anaerobes accounted for 73.3% of the microbial population. Gastroenterologists (94, 37.6%) and general surgeons (52, 20.8%) were the most common prescribers. Hepatobiliary (83, 33.2%), respiratory (58, 23.2%), and intra-abdominal infections (IAI; 34, 13.6%) were the major suspected diagnoses. Blood-culture collection was associated with the use of immunosuppressive agents (OR, 3.48; 95% CI, 1.49–8.99), intrabdominal infection (OR, 0.28; 95% CI, 0.12–0.67), systemic inflammatory response syndrome criteria ≥ 2 (OR, 4.50; 95% CI, 2.25–9.42), and surgical specialty (OR, 0.33; 95% CI, 0.18–0.60). **Conclusions:** More than one-third of patients requiring hospitalization and empiric piperacillin-tazobactam did not undergo blood-culture collection. The finding that blood cultures were less likely to be obtained in patients with suspected IAI requiring hospitalization and by surgical specialties raises a concern regarding suboptimal evaluation. Further assessment of the appropriateness of blood-culture collection in the setting of broad-spectrum antibiotic prescription and tailored promotion of blood-culture collection to surgical specialties may be warranted.

**Disclosures:** S.K.: The author (during graduate school (PhD) was involved in antiviral research relevant to a neglected tropical disease and favipiravir. During this graduate school research, favipiravir was provided by FUJIFILM Toyama Chemical Co. Ltd

